# Doping Existential Despair: Mindful of the Exotic Lure

**DOI:** 10.1007/s12124-024-09839-7

**Published:** 2024-04-13

**Authors:** Luca Tateo

**Affiliations:** Department of Special Needs Education, University of Oslo, Oslo, Postboks 1140 Blindern 0318 Norway

**Keywords:** Tensegrity, Metaphysics, Monadism, Ecology

## Abstract

The paper builds on (von Fircks, E. Integr. psych. Behav. Sci. 2023) article on mindfulness meditation analysed in a Meadian perspective. First, the selective appropriation of some concepts by hegemonic psychology is critically discussed. Then, the consequences of adopting the whole philosophical system of Eastern perspectives are envisaged. Finally, a proposal for a truly ecological shift in the study of self is proposed.

Fircks (2023) proposed an interesting intellectual challenge to psychology, that is to understand how the person can develop and realize new concepts of oneself through the practices of mindfulness meditation whereas Mead’s (2015) theory of the sociogenesis of self and internalized generalized other can provide an insightful integration to describe the process through which mindfulness works. Fircks (2023) illuminated his theoretical discussion with an autoethnographic account in which the accounting “I” is the centre of everything. The interesting hypothesis is that combining the way of relating to thoughts, emotions, and sensations (Kabat-Zinn, 2018) developed through mindful meditation and the idea of the self as hetero and auto-dialogue through signs, one can become aware of the unfolding of self-change and the emergence of a new configuration of the self. Being aware of such as process “help the individual to experience a new I and use altered symbols and encounter his/her social and material environment in new ways” (von Fircks, 2023, 17). The idea of “mindfulness meditation, understood as a set of self-development practices aimed toward moment-to-moment awareness” (du Plessis & Just, 2022, 210) has gained wide popularity in the hegemonic psychology, coming from the peripheral side of what was once non-hegemonic humanistic psychology. The reasons for the success of the idea of mindfulness in hegemonic psychology and its socialization to the general audience is an interesting topic in itself. During the last decades, hundreds thousand of publications, academic and non-academic, methods, therapeutic or self-help, TV-shows and apps have been produced in the name of mindfulness (Fig. 1).

**Fig. 1:**
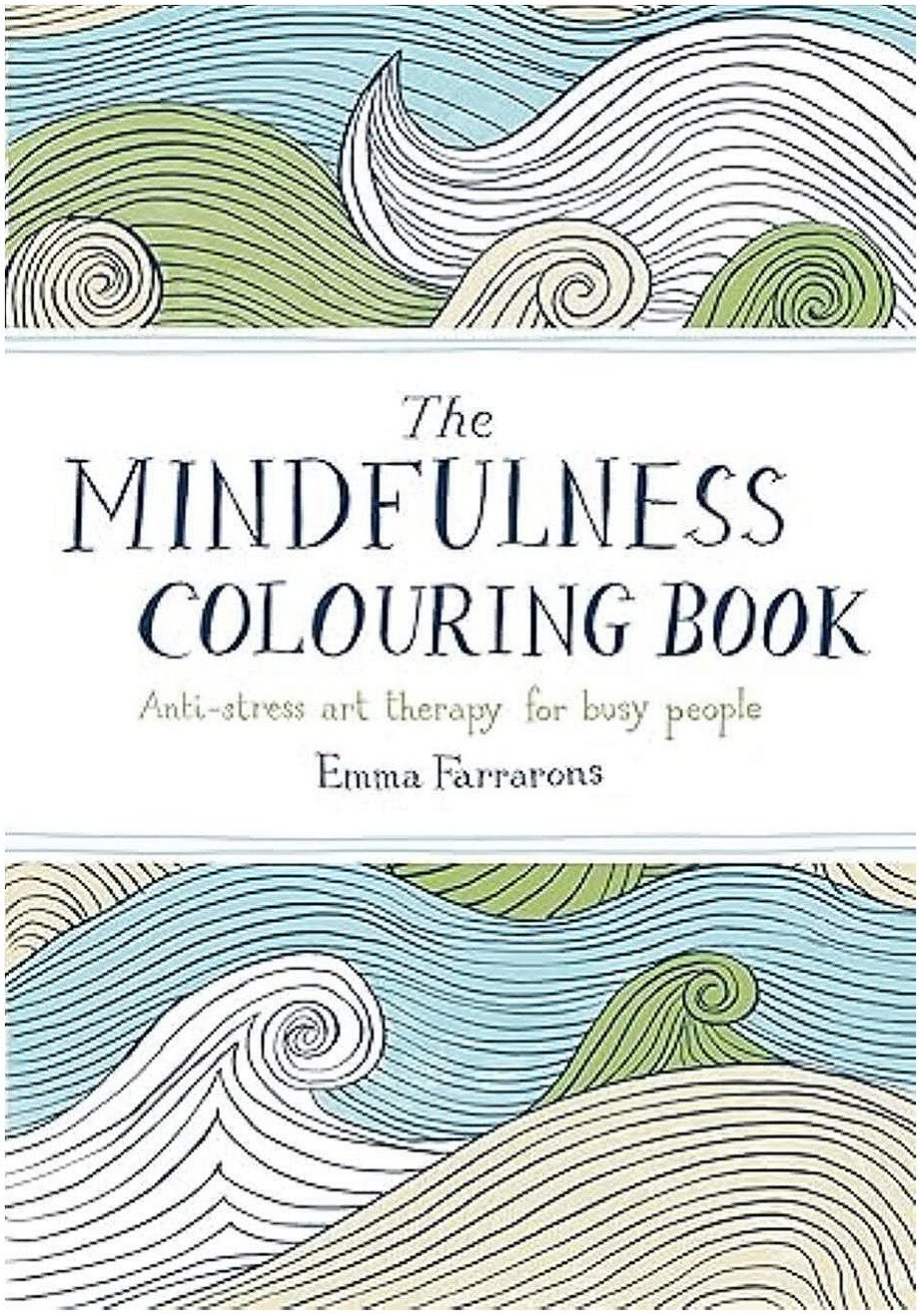
Mindfulness in many fashions (Farrarons, 2015)

It is undoubtable that the fact of mindfulness practice being sold as a product of the Oriental wisdom has contributed to its appeal. Contemporary Orientalism it is indeed an interesting side of the neocolonial and neoliberal endeavour. It is “a system of representations framed by a whole set of forces that brought the Orient into Western learning, Western consciousness, and later, Western empire.” (Said, 1978, 203) As cherry-picking cultural practice, or as cultural appropriation, the use of mindfulness as an Orientalist fashion reflects the typical non-ecological epistemology of hegemonic psychology. Non-ecological means to pretend to separate one element from the whole and transplant it into a completely different ecosystem pretending that it can work in the same way. Yet, one can’t pick a psychology or an anthropology without buying its cosmology and deontology. And frankly, the current technocratic perspective of psychology could hardly buy the deep implications of adopting non-Western cosmologies. It is exactly this conceptual ambivalence that makes the use of mindfulness in psychology and interesting object of reflection.

As von Fircks (2023) wrote, the need for a new perspective on oneself arises from a sense of discomfort or uneasiness. That’s Hesse’s (1998) Westernized interpretation of Siddhartha’s myth. Herman Hesse’s *romans de formation* have been a fundamental reading for thousands of (male) adolescents in Europe, contributing to the spreading of the idealized and domesticated view of Orientalist wisdom. In this sense, such a domestication is not conceptually different from the phenomenon of extreme right fans referring to Bu-shi-do deontology or Wall-street brokers reading Sun-Tzu’s *Art of War*.

Fricks (2023) clearly shows how the interest in mindfulness arose from an existential discomfort and a search for meaning that seems common in the neoliberal performance culture. The utilitarian metaphysics that is hegemonic in the current neoliberal societies is hardly compatible with the relational metaphysics of Eastern philosophies. The former is based on the pursuit of happiness, the latter on the principle of dynamic harmony. The former is ego-centric, and the latter is no-centric (Fig. 2).

**Fig. 2:**
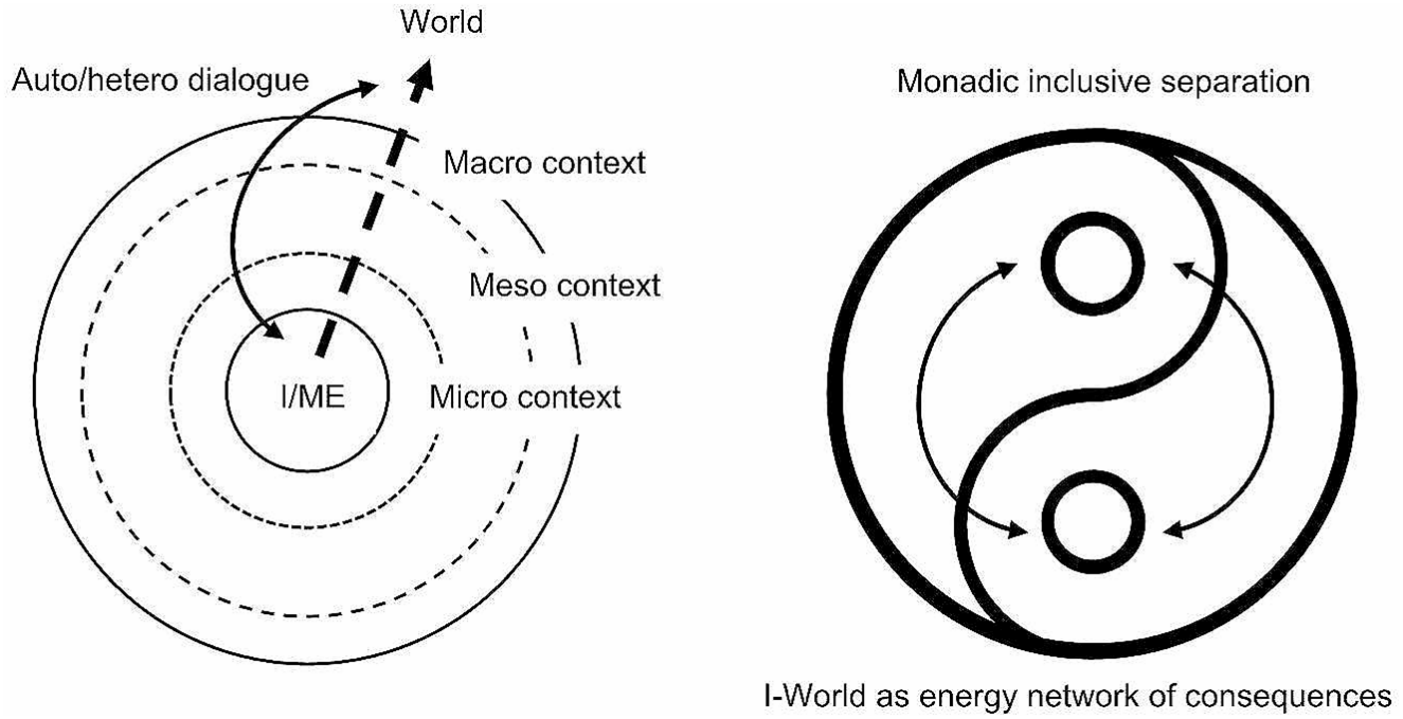
Ego-centric versus no-centric perspectives

The cosmology and metaphysics of hegemonic psychology are based on the constitution and cultivation of the I-World dualism that constitutes the privilege of the controlling subject – over the Other, the nature, over oneself – but also creates the despair of being other-than and of never being fully able to reach-out-there. Although Fricks (2023) aims to build a conceptual system based on a Median-informed mindfulness (p.16), he is still working within the radial ego-perspective of hegemonic psychology. The most difficult thing for a Westerner is to give up the Self as locus of control that has been built with great effort during lifetime, guided by the high value set onto individuation by that specific culture.

Achieving, authorship, authenticity, mentalizing, selfishness, well-being are all understood as achievement of the self through the instrumental (stimulatory or inhibitory) relationship with Otherness. It is the idea of the existence of a “true” self, “the personality that is the individual or personal stance towards a given social situation with its respective demands” (Fircks, 2023, 16). On the contrary, the metaphysics of the philosophical perspectives that emerged from the Indian and China sub-continental areas – Hinduism, Buddhism, Daoism, etc. – are based on the principle that nothing is about “I”, it is rather about the network of consequences that cannot be escaped as any living being – material or spiritual – is part of an interdependent whole. However, it is not by coincidence that Fircks (2023) choose to discuss Daoism, which is the one more Self-oriented among the Eastern philosophies. Nevertheless, the monadic nature of the world is commonly acknowledged by the different spiritual and philosophical perspectives that have been intermingling over the centuries from India to Japan, across China.

When hegemonic psychology tries to selectively adapt and integrate some concepts from Hinduism, Buddhism, or Daoism in its own system it fails to comprehend that those philosophies undermine the very principle of individual psychology. Indeed, they assume the monadic principle of a single type of energy whose manifold sensible manifestations, including the conscious self, are only temporary and superficial. Thus, also the I-World dualism is delusional as well as the idea that there is an active subject in control. For neoliberal individualism this is a very hard bite to swallow and indeed the whole problem of mindfulness in psychology is how to use the meditation techniques imported from the Eastern philosophies and spiritualities to achieving one of the valued personal goals rather than accepting the idea to be one with the world, without egocentric concerns. Used in this way, mindfulness is a sort of exotic “dope” to get through moments of existential dread. The paradox is that by fully adopting the philosophical system in which the idea of mindfulness is grounded, psychology should give up its basic assumptions and accepting to be a “science” of no-ego, no-individualism, no-control, and no-neoliberalism. Imagine what consequences this could have upon whole branches of psychological sciences that deeply rooted in neoliberal capitalist worldview, such as work and organizational psychology, clinical psychology, educational psychology, etc. On the contrary, once extracted from the whole cosmology, anthropology, and deontology of origin, the idea of mindfulness becomes a celebration of the Western vision: self-realization can only come from the inside, no matter what the motivation is – hedonic or eudaemonic – it is all about the “I” that is the centre of the world.

Of course, as Fircks (2023) explained in his autoethnographic account, there is nothing wrong with the strive for self-improvement, self-knowledge, and self-realization. In this sense, he was right in identifying a similar principle in Daoism. However, the most interesting part of its theoretical exploration is the exemplification of the existential tension of the Western neoliberal psyche between the dream of achieving a self-sufficient happiness and the intolerance to suffering that – as one should know from Buddhism – emerges exactly from sticking to the desire of looking for an illusory happiness of an illusory self. The misunderstanding that Fircks (2023) picked from psychological science was that of a self as locomotion across contexts to achieve a new and better place. This metaphor of life course as a journey – that implies other metaphors such as reaching, obstacles, changes of direction, thresholds, etc. - is not an ecological view at all, despite occasional references to open systems. Indeed, there is no self as such – or no self at all? – outside a whole with the world. The relationship between the individual psyches and the world is co-genetic (Tateo, 2106) that is they are codefined through inclusive separation (Fig. 3), in which what one can consider the “I” can only exist as a set of relationships with what is “not-I”.

**Fig. 3:**
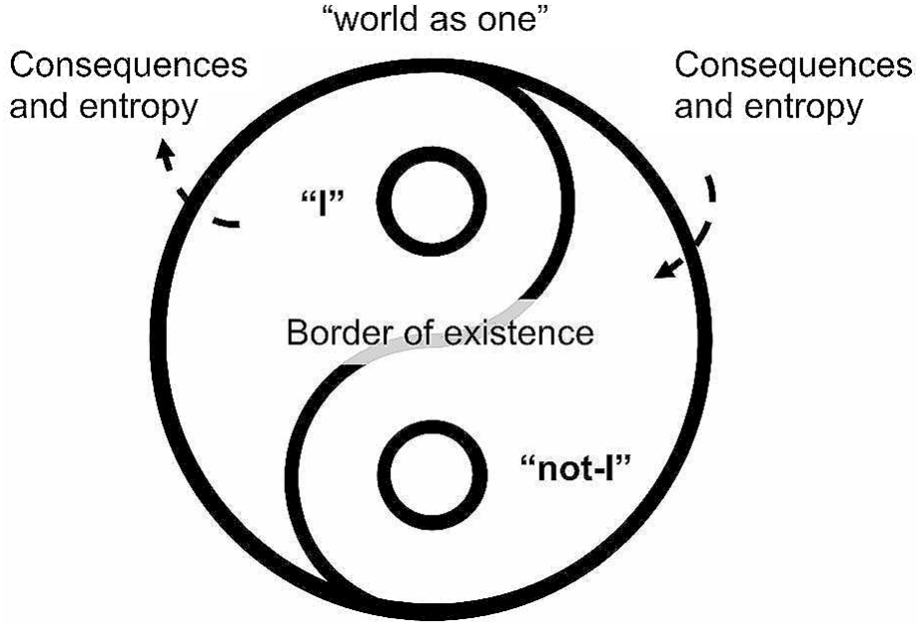
The self as dynamic tension

The metaphysics of Eastern philosophies is deeply ecological in the sense that it conceives the world as a set of relationships, that is a network of consequences and entropy: every action produces consequences, and this is what relates all beings, in all possible directions – past-future, inanimate-animate, human to human, human to non-human, etc. From one egocentric perspective it is not possible to envisage the complex network of consequences, as for the perspective of a single tree in some wood it is impossible to perceive the whole intricated network of the biome. The self is thus the constant and modulating relationship between the “I” as temporary pole of the complementary “non-I” (one could call it the Otherness) whose existence emerges from the dynamic tension of their acting and counter acting (Marsico & Tateo, 2017). Such a de-ontologized self may horrify any Westerner, whose idea if identity is based on the concept of agency. Yet from the perspective of a monadic whole, who is acting on whom?

The role of mindfulness meditation, if one takes the whole system to its consequences, is not that of identifying the “I” who is responsible of its own condition or change (Fricks, 2023), but to become aware of the network of consequences and thus give up the delusion of a stable and clearly bounded self. The difference between the cultural, spiritual, and philosophical traditions is that realizing that the “I” is not a bounded entity, but a dynamic tensegrity of several elements in an ecosystem, created existential dread in the Western philosophical system while produced a deep compassion for suffering and respect for any being in the universe. This is a direct disconfirm of the whole colonial edifice: whereas everything is part of a divine whole, no one is entitled to act as having divine right.

## Data Availability

This manuscript does not report data generation or analysis.
